# Strategic Planning for a Digital Health Innovation Hub at a Saudi Academic Medical Center: Qualitative Case Study

**DOI:** 10.2196/92526

**Published:** 2026-07-15

**Authors:** Mohammed Alshehri, Abdulrahman Mohammed A Altowaijri, Sara Alsubait, Saleh Binsaleh

**Affiliations:** 1 Center of Excellence in Biotechnology Research, King Saud University Medical City, King Saud University, RGSA 8707, Riyadh, Riyadh Region, Saudi Arabia, +966 54 220 5822; 2 QFBA, Northumbria University, Newcastle upon Tyne, England, United Kingdom; 3 Executive Strategic Planning Department Corporate Transformation & Investment Department, King Saud University Medical City, Riyadh, Riyadh Region, Saudi Arabia; 4 Professor, Department of Restorative Dental Sciences, College of Dentistry, Consultant, Dental University Hospital, King Saud University, Riyadh, Riyadh Region, Saudi Arabia; 5 Division of Urology, Department of Surgery, Faculty of Medicine, King Saud University Medical City, King Saud University, Riyadh, Riyadh Region, Saudi Arabia

**Keywords:** digital health, strategic planning, innovation hub, knowledge transfer, Vision 2030, Saudi Arabia, case study, thematic analysis, intellectual property.

## Abstract

**Background:**

Rapid digital transformation is reshaping health care, but many digital initiatives struggle to deliver sustained organizational value when they are introduced as stand-alone technologies rather than as part of an institutional strategy. In Saudi Arabia, Vision 2030 has intensified pressure on academic medical centers to strengthen digital capability, localize innovation, and reduce dependence on externally driven solutions.

**Objective:**

This study aimed to examine organizational factors shaping digital health innovation at King Saud University Medical City (KSUMC) and to develop a preliminary strategic planning framework for a digital health innovation hub tailored to that setting.

**Methods:**

We conducted a qualitative exploratory case study at KSUMC between April and June 2025. Fourteen stakeholders from clinical, administrative, research, governance, educational, and innovation-related roles were recruited using maximum variation purposive sampling. Semistructured interviews were audio-recorded, transcribed verbatim, and analyzed using reflexive thematic analysis. Reporting was guided by the COREQ (Consolidated Criteria for Reporting Qualitative Research). Diffusion of innovation theory and systems thinking informed interpretation, while a context-actor-mechanism-outcome lens was used to examine how institutional conditions shaped innovation processes. No patient data were collected.

**Results:**

Five interrelated themes were identified. First, leadership support existed at a symbolic level, but middle-management translation and risk tolerance were inconsistent. Second, innovation was constrained by workload, fragmented systems, and weak operational support, which meant that project work was often treated as discretionary rather than embedded. Third, knowledge transfer and commercialization pathways were fragmented; participants repeatedly described unclear routes from idea generation to prototyping, regulatory review, and market engagement. Fourth, incentives and innovation capability were misaligned with institutional expectations, particularly for clinicians and trainees. Fifth, Vision 2030 created strategic legitimacy and momentum, but participants also cautioned that an overreliance on consultant-led or vendor-led approaches could weaken internal capability building. These findings informed a preliminary framework centered on governance, knowledge transfer, partnership structures, workforce development, and phased implementation rather than a validated institutional model.

**Conclusions:**

At KSUMC, digital health innovation is shaped not only by technology availability but also by organizational culture, intermediary structures, governance design, and the extent to which innovation work is made operationally feasible. The framework proposed here should therefore be understood as a preliminary planning model derived from one qualitative case study. Its main contribution is to specify how knowledge transfer, commercialization, and institutional capability building can be integrated into a digital health strategy within a Saudi academic medical center.

## Introduction

### Background

Digital technologies such as telemedicine, mobile health, AI, and data analytics have significant potential to improve care quality, efficiency, and access. However, digital tools do not create transformation on their own. Recent work in digital health strategy and implementation has emphasized that sustainable change depends on leadership, governance, interoperability, workforce readiness, and alignment between technology choices and service priorities [1-5]. In practice, organizations often struggle because digital projects are introduced through fragmented pilots, vendor-led procurement, or ad hoc local enthusiasm rather than through coherent institutional planning [1,2,4,5].

This issue is particularly important in academic medical centers, where clinical care, research, education, and innovation activity intersect. Studies from other contexts have shown that hospitals and university-affiliated health systems often face similar problems when trying to institutionalize digital innovation. These include uncertainty over who owns digital strategy, how responsibilities should be distributed across departments, how digital initiatives should be governed, and how promising ideas can move from early development into routine operational or commercial use [2,3,6,7]. Related work has also highlighted recurring barriers, such as siloed information systems, resistance to organizational change, limited protected time, weak cross-sector collaboration, and the absence of formal structures to support knowledge transfer and implementation [4,6,7]. Taken together, these studies suggest that the challenge is not simply adopting digital tools but creating organizational conditions that allow innovation to be coordinated, evaluated, and sustained.

In Saudi Arabia, these questions sit within the broader policy agenda of Vision 2030 and the Health Sector Transformation Program, both of which place strong emphasis on modernization, digital capability, and local innovation capacity [8-12]. King Saud University Medical City (KSUMC) is a major academic medical center operating within this policy environment. As part of its institutional development, it has shown growing interest in creating an innovation hub able to support digital health projects, knowledge transfer, and intellectual property (IP) management [9,13,14]. This makes KSUMC a useful case through which to examine how broader strategic ambitions are interpreted at the organizational level.

The World Health Organization (WHO) Global Strategy on Digital Health provides a broad systems framework for leadership, governance, standards, and workforce development [15]. The Centers for Disease Control and Prevention (CDC) Global Digital Health Strategy was considered alongside it because it adds a more operational emphasis on data use, public health infrastructure, and digital workforce enablement [16]. Although these frameworks are valuable, they operate at a relatively high level and do not in themselves explain how an academic medical center should structure responsibilities, prioritize investments, connect innovation with implementation, or build internal capability in a resource- and context-sensitive way. Moreover, while studies from other settings have identified common barriers and enablers, the literature remains limited on how such insights can be adapted to academic medical centers in the Gulf region, where policy priorities, organizational arrangements, and innovation ecosystems differ from those in the North American and European settings that dominate the literature [17-19].

The principal knowledge gap concerns not whether digital health matters, but rather how a locally grounded and organizationally feasible institutional strategy can be developed for an academic medical center in a setting where innovation capability is still emerging and where commercialization, workforce development, and knowledge transfer must be addressed alongside service delivery. This study addresses that gap by examining the KSUMC context and developing a strategic planning framework for a digital health innovation hub that is informed by international guidance but rooted in local organizational realities.

### Study Aim and Research Questions

This study aimed to develop a strategic planning framework for a digital health innovation hub at KSUMC. Specifically, it examined which organizational and systemic factors shape digital health innovation at KSUMC, how global digital health planning frameworks can be interpreted in a Saudi academic medical center, and which components should be prioritized in a local framework that supports innovation, knowledge transfer, and IP commercialization.

## Methods

### Study Design and Reporting Framework

We undertook a qualitative exploratory case study at KSUMC between April and June 2025. A case study design was appropriate because the study sought to examine digital health innovation within its real-world organizational context rather than isolate it from that context [20]. The study was reported with reference to the COREQ (Consolidated Criteria for Reporting Qualitative Research) checklist [21].

### Sampling Strategy and Participants

Maximum variation purposive sampling was used to recruit information-rich participants whose roles placed them at different points in the innovation ecosystem [22]. The aim was not statistical representation but conceptual breadth across clinical practice, research, governance, training, partnerships, and innovation management. Eligibility criteria were employment at KSUMC or a closely affiliated university or medical-city function; direct involvement in innovation-related, digital, research, governance, commercialization, or training activities; and sufficient institutional familiarity to comment on organizational processes. The target experience threshold was 2-3 years at KSUMC or equivalent familiarity with the system [22,23].

Potential participants were identified by the lead author through institutional role mapping against the target domains (clinical practice, research, governance, training, partnerships, and innovation management). Invitations were sent by email, supplemented by internal professional networks. Although the sampling frame was purposive, recruitment also had a pragmatic, convenience-based element given the single-site setting and the limited number of stakeholders occupying the relevant roles; this is acknowledged as a limitation. We approached approximately 16 stakeholders, and 14 agreed to participate. No enrolled participant withdrew after interview, and no repeat interviews were conducted. Because this was a single-site study and some roles were highly identifiable, participant characteristics are reported at a broad, nonidentifying level. Age and gender were not emphasized in reporting to reduce deductive disclosure risk in a relatively small organizational setting. At a nonidentifying aggregate level, the sample nonetheless spanned a range of seniority and functions: senior leadership (including unit, research, and partnership directors), middle-management and governance roles, and operational staff directly engaged in innovation, commercialization, and training activities, distributed across the 5 stakeholder domains summarized in Table 1.

**Table 1.** Broad participant characteristics reported at a nonidentifying level.

Stakeholder domain	Roles represented
Clinical and clinician-academic perspectives	Clinical consultant, professor-consultant involved in device development, and senior clinically engaged stakeholders
Research and research governance	Research director, research governance officer, and research-support stakeholders
Innovation, commercialization, and partnerships	Innovation unit leader; head of innovation partnerships; and staff supporting innovation programs, hackathons, and commercialization pathways
Education and training	Academic training program director
Senior strategic and administrative perspectives	Executive strategic planning leaders and cross-institutional administrative stakeholders involved in institutional strategy and policy

### Data Collection

Semistructured interviews were conducted one-to-one, primarily through secure videoconferencing, with some flexibility in modality depending on participant availability. Interviews generally lasted 40 to 60 minutes, with a few extending longer when participants wished to elaborate. Only the participant and researcher were present during the interview. The interview guide covered current innovation practices, knowledge transfer, commercialization, barriers and enablers, external partnerships, and views on the design of an innovation hub. Probing questions were used to obtain examples and clarify how participants understood organizational processes. Interviews were audio-recorded and transcribed verbatim, and field notes were used to capture contextual impressions and emerging analytic ideas. Transcripts were not routinely returned to participants for correction.

The interview guide was informed by the literature review and piloted before full data collection. Following the pilot, the guide was refined to improve the clarity of the questions and their alignment with the theoretical framing. In addition to interview data, the researcher reviewed a small amount of contextual documentary material, such as organizational descriptions and strategy-related information. This material was used only to orient the researcher to the institutional context and to refine interview questions and interpretation; it was not formally coded or analyzed thematically, and interviews remained the sole primary data source.

The lead author conducted all interviews and led the primary analysis. No formal relationship was established with participants specifically for the study before recruitment, although some participants may have been aware of the researcher through the wider institutional or professional context. Participants were informed that the researcher was undertaking the study as an academic project focused on innovation, knowledge transfer, and digital health strategy at KSUMC. The researcher approached the study from an interpretivist position, which brought useful contextual sensitivity but also created a risk of privileging strategic or solution-oriented interpretations. To manage this, the researcher used a semistructured guide, maintained analytic notes during coding, discussed emerging interpretations with the supervisor and a second reader, and sought to preserve disconfirming or critical viewpoints rather than smooth them into consensus. The analysis therefore treated the data as accounts of organizational experience rather than as neutral institutional facts.

### Data Analysis

Data were analyzed using reflexive thematic analysis [24,25]. Analysis followed the familiarization, coding, theme generation, review, definition, and write-up phases, but iteratively rather than in a rigid sequence [24,25]. Initial coding remained close to participants' language. Codes were then grouped into candidate themes that were repeatedly checked against the full dataset. A second coder reviewed the full coding framework and thematic definitions as they evolved, acting as a critical reviewer rather than independently coding the transcripts; consistent with reflexive thematic analysis, intercoder reliability was not sought. Differences in interpretation were resolved through discussion focused on fit with the data, conceptual coherence, and the scope of each theme. Coding was managed manually rather than through specialist qualitative analysis software. Formal participant checking of the final thematic findings was not undertaken within the study timeline.

Theoretical frameworks informed interpretation rather than being imposed as a deductive coding template. Diffusion of innovation theory was used to interpret issues such as leadership signaling, championing, resistance, and adoption pathways [26]. The context-actor-mechanism-outcome (CAMO) lens helped examine how organizational conditions, such as bureaucracy, workload, and fragmented authority, shaped what participants and units were able to do [27]. Systems thinking supported interpretation of interdependencies across governance, infrastructure, incentives, and partnerships rather than treating them as isolated barriers [2,27]. Trustworthiness was supported through an audit trail of coding decisions, reflexive memoing, peer discussion, and the use of thick description in the reporting of findings [28]. Sample sufficiency was judged pragmatically using the specificity of the study aim, the expertise of participants, and the recurrence of patterned meanings rather than a fixed numerical threshold [23,29].

### Ethical Considerations

Two separate authorizations governed this study. Organizational consent from KSUMC constituted institutional permission to access the site and approach participants (gatekeeper authorization) and did not constitute a formal research ethics review. Preliminary verbal institutional authorization to commence was granted before the first interviews in April 2025, and this organizational consent was subsequently formalized in writing on May 25, 2025; interviews were conducted between April and June 2025. Formal research ethics approval, by contrast, was provided by the Northumbria University Research Ethics Committee and granted on July 4, 2025; because data collection had been completed before this date, the Committee’s approval was obtained retrospectively. No reference number was assigned, as per institutional procedures. All participants provided informed consent before the interview, either in written form or as recorded verbal consent documented at the start of the interview. Participation was voluntary, and participants were reminded that they could decline any question or withdraw at any time.

Confidentiality was protected through coded participant identifiers, careful removal or generalization of identifying details, secure storage of audio files and transcripts on password-protected devices, and aggregate reporting of potentially sensitive institutional observations. The study did not collect patient data. No participant compensation was provided. Participants were also informed that they could request a summary of the study findings after completion.

## Results

### Overview of Themes

Five interrelated themes captured how digital health innovation was experienced at KSUMC: leadership and culture, resource and operational constraints, knowledge exchange and IP pathways, incentives and capability development, and the external policy environment. Together, these themes suggested that innovation at KSUMC was not blocked by a single missing input. Rather, participants described a system in which leadership endorsement, governance rules, funding, training, partnerships, and commercialization support needed to function together. This systems view became important in the later development of the framework. The themes, illustrative subthemes, and representative quotations are summarized in Table 2.

**Table 2.** Themes, subthemes, and illustrative quotations.

Theme	Illustrative subthemes	Illustrative quotations
Leadership and culture	Symbolic support from senior leaders, weak middle-management translation, and low tolerance for failure	“Leadership is supportive in principle, but systems and policies are not yet aligned to act.”
Resource and operational constraints	High workload, lack of protected time, fragmented systems, and weak project support	“Innovation feels like something separate from the day-to-day pressures of patient care.”
Knowledge exchange and intellectual property pathways	No clear route from idea to implementation, weak technology transfer coordination, and informal rather than institutional collaboration	“There’s no clear pathway for idea development and research.”
Incentives and capability development	Limited innovation literacy, promotion and reward systems favor publications, and scarce training and mentorship	“Innovation should not be an extra, it should be part of how we train future clinicians to think, solve, and lead.”
External policy environment	Vision 2030 creates momentum, risk of consultant-led dependence, and partnerships require internal readiness	“We should be co-developing solutions with industry, not just serving as research sites.”

### Leadership and Innovation Culture

Participants described leadership as necessary but insufficient. Several stakeholders acknowledged visible endorsement from senior leaders, innovation boot camps, hackathons, or committee activity. At the same time, they emphasized that support often weakened once projects moved into approval pathways or cross-departmental implementation. One participant noted that leadership was “supportive in principle, but systems and policies are not yet aligned to act.” Another argued that “culture first” was the right principle because structures alone would not change behavior. These comments suggest that leadership at KSUMC currently functions more as symbolic authorization than as a consistently enabling implementation mechanism.

Interpreted through diffusion of innovation theory, this theme points to problems not at the awareness stage but at the persuasion, decision, and implementation stages [26]. New ideas may be welcomed rhetorically, yet institutional actors do not encounter the low-friction pathways needed to test them. Participants repeatedly located this tension in middle management, where compliance demands, unclear authority, and risk aversion translated top-level support into slow or inconsistent operational action.

From a systems perspective, culture was not described as a purely attitudinal issue. It was tied to how authority is distributed, whether experimentation is protected, and whether innovators receive visible recognition. Participants therefore framed leadership not only as executive sponsorship but also as the ability to make innovation legitimate within everyday work.

### Resource and Operational Constraints

Resource constraints extended beyond lack of money. Participants spoke about high clinical workload, limited protected time, insufficient project management support, and fragmented digital systems that made innovation work difficult to sustain. A clinician commented that “innovation feels like something separate from the day-to-day pressures of patient care,” while others noted that projects were often layered on top of normal duties rather than built into service priorities.

These accounts indicate that innovation at KSUMC is still treated as exceptional work rather than routine organizational work. In CAMO terms, the context of workload pressure and fragmented operational systems narrowed what actors could realistically do, even when they were motivated [27]. This helps explain why participants frequently linked innovation failure to follow-through rather than to idea generation. The barrier was not an absence of ideas, but a weak bridge between idea ownership and operational execution.

Participants also tied these constraints to digital infrastructure. Fragmented systems reduced the practical value of innovation, especially when teams could not easily access data, coordinate approvals, or test solutions in live service environments. The problem was therefore sociotechnical: both workflow conditions and system architecture limited implementation.

### Knowledge Exchange and IP Management

A strong pattern across interviews was the absence of a clear institutional route from idea generation to prototyping, validation, governance review, IP protection, and commercialization. A clinical participant summarized this simply: “There’s no clear pathway.” Research and innovation stakeholders similarly described a fragmented process in which collaboration was often informal, highly dependent on individual champions, and weakly connected to market or regulatory realities. Participants highlighted gaps in legal support, project navigation, prototype development, and technology transfer capacity.

This theme moved the analysis beyond a generic statement that knowledge transfer is important. What participants described was a missing intermediary layer. Informal networks existed, and some successful examples were mentioned, such as device development or a digital risk calculator used in clinical pathways. However, these successes were treated as proof-of-concept exceptions rather than evidence of a stable institutional pipeline. In systems terms, the organization had islands of innovation but limited connective tissue between them [2,27].

The commercialization issue was therefore not simply low patenting knowledge. Participants repeatedly connected weak commercialization outcomes to the lack of coordinated actor relationships among researchers, clinicians, governance teams, industry, and funders. This interpretation supports the inclusion of knowledge transfer and IP support as central rather than peripheral components of the proposed framework.

### Incentives, Capacity, and Workforce Development

Participants described strong intrinsic motivation to improve care, solve local problems, or gain recognition. However, they also emphasized that institutional incentives were poorly aligned with innovation work. Publication outputs were perceived as more visible and more rewarded than implementation, prototyping, partnership building, or commercialization. One participant observed that trainees were encouraged into research, but mostly in passive forms, while another stressed that “innovation should not be an extra, it should be part of how we train future clinicians to think, solve, and lead.”

This theme suggests that innovation capability at KSUMC is limited by both skill development and reward design. Participants requested training in design thinking, entrepreneurship, regulatory pathways, IP, and market assessment, along with mentorship and protected time. The findings therefore point to a dual gap: people need practical innovation literacy, but they also need institutional signals that such work counts. Without both, innovation remains dependent on unusually persistent individuals rather than becoming a reproducible organizational capability.

Diffusion theory helps clarify why this matters. Adoption depends partly on whether innovation appears compatible with current professional roles and reward structures [26]. At KSUMC, participants often described innovation as adjacent to formal work rather than integrated into it. This made participation harder for clinicians, trainees, and early-career researchers.

### External Policy Environment

Participants viewed Vision 2030 as a powerful source of legitimacy for innovation. It created strategic momentum for localization, private-sector engagement, and institutional modernization. At the same time, participants worried that external consultants, vendors, or short-term project partnerships could substitute for internal capability development [30]. One partnership leader stated that KSUMC should be “co-developing solutions with industry, not just serving as research sites.” Another recommended starting with small pilot models and scaling only after demonstrating feasibility.

This theme is analytically important because it shows that the external environment operates in two directions. National policy can enable innovation by opening funding channels and giving organizations permission to act. But it can also incentivize superficial compliance if institutions respond mainly through outsourced strategy production or technology acquisition. In CAMO terms, the macropolicy context creates opportunity, yet the mechanism through which that opportunity translates into local capability depends on institutional readiness and internal coordination [27].

The implication is that external partnerships should complement, not replace, local learning. Participants consistently preferred collaborative models that built local capability in governance, prototyping, commercialization, and implementation. This interpretation shaped the more cautious, phased approach taken in the framework discussion below.

## Discussion

### Principal Findings

This study adds to the digital health strategy literature by moving from broad strategic principles to a single-site organizational account of how those principles are constrained, translated, or interrupted inside an academic medical center. Its contribution is therefore not the invention of completely new strategic components. Rather, it is the contextually grounded combination of digital health strategy, knowledge transfer, and commercialization concerns into one institutional planning problem for KSUMC. Participants did not simply ask for more technology. They repeatedly identified the need for intermediary structures, clearer pathways, protected time, and implementation-capable governance. That combination is what gives the framework its local distinctiveness.

The findings are consistent with recent work showing that digital transformation depends on organizational readiness, and not just on technology acquisition [4,5]. However, the present study extends that discussion by highlighting the specific importance of knowledge transfer and IP pathways in an academic medical center. In KSUMC, the challenge was not only digital adoption; it was also how ideas move from clinical need or research insight into validated, governable, and potentially scalable solutions. This is why the proposed framework places commercialization support, partnership design, and capability building alongside more familiar governance and infrastructure issues.

The framework should be understood as a preliminary planning model informed by one qualitative case study, not as a validated tool ready for institutional rollout. The data support the identification of planning priorities and design principles, but they do not by themselves validate the effectiveness of any specific organizational structure. Some proposed elements, such as a steering group, a knowledge transfer and IP function, or phased pilot governance, were grounded strongly in participant accounts. Other elements, such as commercialization reinvestment mechanisms or semiautonomous hub design, are better understood as recommendations derived from the findings in combination with the wider literature [5-7,31].

This distinction matters because participants also described major feasibility constraints. Bureaucratic approval pathways, limited time, funding gaps, fragmented authority, and weak intermediary support all suggest that a full-scale hub model may be unrealistic if introduced as a single institutional reform. A more feasible interpretation is phased implementation: first, clarify governance routes and create an innovation navigation function; second, build pilot funding, training, and partnership support; and third, expand toward more formal commercialization and incubation capacity once proof-of-concept successes and trust have accumulated.

WHO and CDC strategy guidance remained useful in this analysis, but mainly as interpretive scaffolding rather than as direct solutions [15,16]. WHO guidance aligns closely with the study’s emphasis on governance, standards, interoperability, and workforce development [15]. The CDC strategy adds a practical focus on data use and workforce enablement, which resonated with participants’ concerns about fragmented systems and weak digital capability [16]. However, the findings suggest that global strategy frameworks need local organizational translation. At KSUMC, questions about who owns a project, how quickly it can move through governance, and where innovators go for legal or IP advice were more immediate than abstract agreement with national digital goals. This is consistent with systems-based accounts showing that health care organizations often struggle less with strategic aspiration than with distributed execution [2,5].

### Comparison With Other Models

Existing frameworks, such as the Consolidated Framework for Implementation Research [32], ExpandNet, and the Hybrid Health Care Quality Assessment model offer guidance for implementation and scaling of innovations [3,27]. However, they are not tailored specifically to digital health strategic planning in academic medical centers. Linnéusson et al [2] noted that hospitals often struggle with defining digital strategies, deciding which responsibilities should be centralized or decentralized, and identifying the system conditions necessary to enable successful transformation. Our framework addresses these questions by combining centralized governance (an innovation steering committee and knowledge transfer office) with distributed innovation teams and by specifying prerequisites, such as interoperable infrastructure and protected time.

### Implications for KSUMC

For KSUMC, the most immediate implication is that digital health innovation should be organized as a pathway problem. Participants repeatedly described unclear transitions between ideation, governance, validation, partnership development, and commercialization. This suggests 3 short-term priorities: a visible governance route for innovation projects; dedicated liaison support able to navigate ethics, legal, data, and IP questions; and small-scale pilot resources that reduce the burden on clinicians and early innovators.

A second implication concerns incentive alignment. Training, promotion criteria, and recognition systems currently appear to privilege conventional academic outputs more strongly than innovation implementation. Participants did not frame this as opposition to research. Instead, they highlighted the need for complementary reward systems that recognize translation, prototyping, partnership building, and operational problem solving. Embedding innovation literacy into training programs and creating protected time for selected pilot work would therefore address both capability and legitimacy.

A third implication concerns partnership design. Participants wanted stronger industry engagement but not on purely outsourced terms. The preferred model was selective co-development around local clinical problems, supported by clearer legal templates, data governance, and role definition. In practice, this means starting with bounded pilots and partnership structures that build internal learning rather than importing complete external solutions. Taken together, these priorities suggest a sequenced agenda: in the short term, establishing a visible governance route and innovation navigation support; in the medium term, building pilot funding, training, and partnership capacity; and over the longer term, developing more formal commercialization and incubation capability as early successes accumulate.

**Figure 1.**
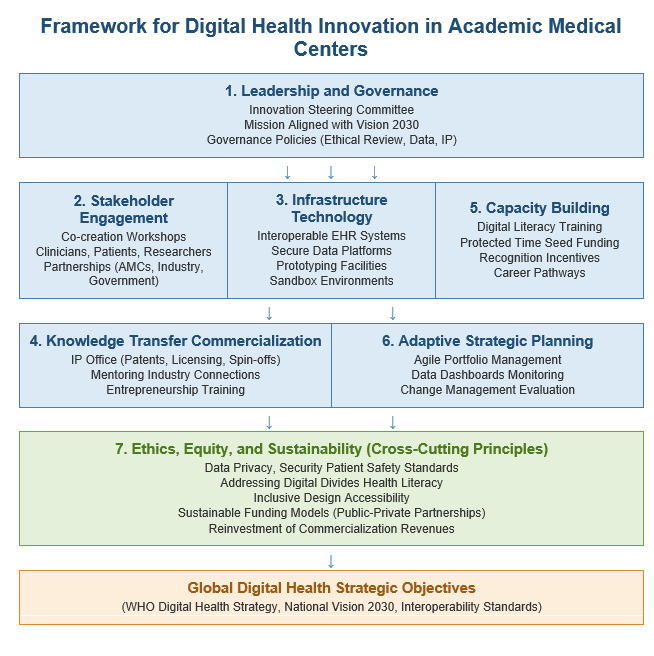
Preliminary strategic planning framework for a digital health innovation hub at King Saud University Medical City, showing the relationships among its core components, governance, knowledge transfer and intellectual property (IP) support, partnership structures, and workforce capability, and their grounding in global digital health strategic objectives. AMC: academic medical center; EHR: electronic health record; WHO: World Health Organization.

Figure 1 outlines the relationships among these components and highlights their grounding in global digital health strategic objectives. The framework was not adopted from any single existing model; it was developed through an interpretive synthesis in which the empirical themes defined its core components and international strategy guidance (notably the WHO and CDC frameworks) served as organizing scaffolding rather than as a prescriptive structure.

### Limitations

This study drew on perspectives across multiple institutional functions, allowing innovation to be analyzed as an organizational system rather than only as a clinical or technical issue, and the interview material supported interpretive analysis of why innovation stalls rather than only a list of barriers. These strengths notwithstanding, the study has several limitations. It was conducted at a single academic medical center, so the findings are context-bound. The sample was appropriate for an exploratory qualitative study, but it was not designed to be statistically representative. Some participant characteristics were deliberately generalized to protect anonymity in a relatively small organizational setting. Several further limitations should be noted. Because participants were organizational insiders reflecting on their own institution, their accounts may have been subject to social desirability bias, with some reluctance to characterize internal processes critically. The sample may also have skewed toward stakeholders holding stronger or more favorable views on innovation, given that recruitment centered on individuals already engaged in innovation-related roles. The study did not capture patient or frontline clinical perspectives, which may narrow the breadth of the organizational picture and omit views that could qualify the stakeholder accounts presented here. Finally, the study relied on a single primary data collection method, namely semistructured interviews supplemented only by limited contextual documentary material, so the absence of methodological triangulation may constrain the robustness of the findings. Member checking of the final thematic findings was also not undertaken within the study timeline, which may further limit interpretive validation. In addition, the framework has not yet been tested through implementation, stakeholder workshops, or longitudinal evaluation. Transferability therefore lies in the conceptual patterns described here, not in assuming that the exact structure would suit every institution.

### Conclusions

Digital health innovation at KSUMC is shaped by the interaction of leadership, organizational culture, workflow pressure, intermediary support, incentive structures, and the external policy environment. The study therefore suggests that strategic planning for a digital health innovation hub should begin with governance clarity, knowledge transfer pathways, partnership support, and workforce capability rather than with technology procurement alone. The framework proposed here is best understood as a preliminary, context-specific planning model that can guide further institutional refinement, stakeholder testing, and phased implementation. The immediate next step is to validate and refine the framework through stakeholder validation, feasibility testing, and evaluation of phased implementation before it is adopted in practice.

## Supplementary material

10.2196/92526 Checklist 1 COREQ checklist.

## Abbreviations

CAMO: context-actor-mechanism-outcome
CDC: Centers for Disease Control and Prevention
COREQ: Consolidated Criteria for Reporting Qualitative Research
IP: intellectual property
KSUMC: King Saud University Medical City
WHO: World Health Organization

## References

[1] Greenhalgh T, Wherton J, Papoutsi C (2017). Beyond adoption: a new framework for theorizing and evaluating nonadoption, abandonment, and challenges to the scale-up, spread, and sustainability of health and care technologies. J Med Internet Res.

[2] Linnéusson G, Andersson T, Kjellsdotter A, Holmén M (2022). Using systems thinking to increase understanding of the innovation system of healthcare organisations. J Health Organ Manag.

[3] Scarbrough H, Kyratsis Y (2022). From spreading to embedding innovation in health care: implications for theory and practice. Health Care Manage Rev.

[4] Brommeyer M, Whittaker M, Liang Z (2024). Organizational factors driving the realization of digital health transformation benefits from health service managers: a qualitative study. J Healthc Leadersh.

[5] Cresswell K, Williams R (2024). Essential strategic principles for planning and developing digitally enabled interventions in health and care settings. BMC Health Serv Res.

[6] Bailey AG, Reingold BM, Johnson JD, O’Connor AC (2025). Paths towards commercialization: evidence from NIH proof of concept centers. J Technol Transf.

[7] Haidegger TP, Galambos P, Tar JK (2024). Strategies and outcomes of building a successful university research and innovation ecosystem. Acta Polytech Hung.

[8] Mani ZA, Goniewicz K (2024). Transforming healthcare in Saudi Arabia: a comprehensive evaluation of Vision 2030’s impact. Sustainability.

[9] Suleiman AK, Ming LC (2025). Transforming healthcare: Saudi Arabia’s Vision 2030 healthcare model. J Pharm Policy Pract.

[10] Alfahad AH, Alabbas YS, Alabbas HS (2024). Evaluating the impact of Saudi Vision 2030 on healthcare investment: a comprehensive review of progress and future directions. J Ecohuman.

[11] Alsari SM, Alzamanan MM, Salem HA (2023). The impact of Vision 2030 on the healthcare system in Saudi Arabia. J Int Crisis Risk Commun Res.

[12] Al-Anezi FM (2025). Challenges of healthcare systems in Saudi Arabia to delivering Vision 2030: an empirical study from healthcare workers perspectives. J Healthc Leadersh.

[13] Alnasser Alqahtani SS (2015). King Saud University’s strategic plan formulation (KSU 2030). King Saud University.

[14] Alnasser A, Al Ghofaili AY, Almushaiti MA, AlZaidan ZI (2024). Navigating digital transformation in alignment with Vision 2030: a review of organizational strategies, innovations, and implications in Saudi Arabia. J Knowl Learn Sci Technol.

[15] (2021). Global strategy on digital health 2020-2025. World Health Organization.

[16] (2022). CDC global digital health strategy. Centers for Disease Control and Prevention.

[17] Haukipuro L, Väinämö S, Virta V, Perälä‐Heape M (2024). Key aspects of establishing research, knowledge, and innovation‐based hubs as part of the local innovation ecosystem. R D Manag.

[18] Benam CH, Baler G, Duke R (2021). Fostering innovation at academic medical centers: the case of University of Colorado Anschutz Medical Campus. J Clin Transl Sci.

[19] Neary K, Iakovleva T, Oftedal EM, Bessant J (2025). Meeting the Inclusion Challenge in Innovation: Giving Voice to Users.

[20] Crowe S, Cresswell K, Robertson A, Huby G, Avery A, Sheikh A (2011). The case study approach. BMC Med Res Methodol.

[21] Tong A, Sainsbury P, Craig J (2007). Consolidated criteria for reporting qualitative research (COREQ): a 32-item checklist for interviews and focus groups. Int J Qual Health Care.

[22] Palinkas LA, Horwitz SM, Green CA, Wisdom JP, Duan N, Hoagwood K (2015). Purposeful sampling for qualitative data collection and analysis in mixed method implementation research. Adm Policy Ment Health.

[23] Malterud K, Siersma VD, Guassora AD (2016). Sample size in qualitative interview studies: guided by information power. Qual Health Res.

[24] Braun V, Clarke V (2006). Using thematic analysis in psychology. Qual Res Psychol.

[25] Braun V, Clarke V (2021). One size fits all? What counts as quality practice in (reflexive) thematic analysis?. Qual Res Psychol.

[26] Zhang X, Yu P, Yan J, Ton A M Spil I (2015). Using diffusion of innovation theory to understand the factors impacting patient acceptance and use of consumer e-health innovations: a case study in a primary care clinic. BMC Health Serv Res.

[27] Kwamie A, Ha S, Ghaffar A (2021). Applied systems thinking: unlocking theory, evidence and practice for health policy and systems research. Health Policy Plan.

[28] Nowell LS, Norris JM, White DE, Moules NJ (2017). Thematic analysis: striving to meet the trustworthiness criteria. Int J Qual Methods.

[29] Hennink MM, Kaiser BN, Marconi VC (2017). Code saturation versus meaning saturation: how many interviews are enough?. Qual Health Res.

[30] Ansari D, Werenfels I (2023). Shadow players: Western consultancies in the Arab world: how multinational consulting firms shape public policy.

[31] Desai M, Tardif-Douglin M, Miller I (2024). Implementation of Agile in healthcare: methodology for a multisite home hospital accelerator. BMJ Open Qual.

[32] Damschroder LJ, Reardon CM, Widerquist MA, Lowery J (2022). The updated Consolidated Framework for Implementation Research based on user feedback. Implement Sci.

